# Navigating the Eloquent Brain: A Multicenter Study on the Safety and Efficacy of Symptomatic Cavernoma Resection

**DOI:** 10.3390/brainsci16070747

**Published:** 2026-07-15

**Authors:** Hojka Rowbottom, Tomaž Velnar, Janez Ravnik, Ninna Kozorog, Tomaž Šmigoc

**Affiliations:** 1 Department of Neurosurgery, University Medical Centre Maribor, 2000 Maribor, Slovenia; hojka.rowbottom@ukc-mb.si (H.R.); janez.ravnik@ukc-mb.si (J.R.);; 2 Department of Neurosurgery, University Medical Centre Ljubljana, 1000 Ljubljana, Slovenia; tomaz.velnar@kclj.si

**Keywords:** cerebral cavernous malformation, eloquent brain regions, microsurgical resection, intraoperative neuromonitoring, seizure control, awake craniotomy

## Abstract

**Highlights:**

**What are the main findings?**

- **High rate of complete resection:** In this descriptive retrospective cohort, complete microsurgical resection of symptomatic cavernous malformations in eloquent brain tissue was achieved, alongside an 80% total removal rate of the surrounding hemosiderin rim.
- **Complete long-term seizure freedom:** At a maximum 48-month follow-up, 100% of patients achieved complete seizure control, allowing nearly half (44.4%) of the cohort to safely discontinue antiepileptic medication.

**What are the implications of the main findings?**

- **Feasibility in eloquent regions:** These observations suggest that careful microsurgical resection of lesions in functionally critical regions can be performed with minimal permanent morbidity, enabling 77.8% of patients in our series to work following surgery.
- **Role of multimodal adjuncts:** The favorable functional outcomes observed support the utility of combining anatomical neuronavigation, structural intraoperative ultrasound, and functional neuromonitoring (including awake craniotomy) to safeguard critical brain networks.

**Abstract:**

**Background/Objectives**: Surgical management of cerebral cavernous malformations (CCMs) within eloquent brain regions presents a high risk of neurological deficits. This study describes the clinical outcomes and technical feasibility of microsurgical resection for symptomatic eloquent CCMs, detailing the integration of advanced intraoperative adjuncts aimed at optimization of seizure control and functional preservation. **Methods**: We conducted a retrospective multicenter analysis of nine adult patients who underwent microsurgical resection for symptomatic eloquent CCMs between January 2021 and December 2025 across two tertiary centers in Slovenia. Intraoperative modalities included 100% neuronavigation, 55.6% intraoperative ultrasound, 77.8% intraoperative neuromonitoring (IONM) with motor and somatosensory evoked potentials, and 22.2% awake craniotomies. **Results**: Seizures were the primary clinical presentation in 77.8% of patients (66.7% medically refractory), and the overall hemorrhage rate was 66.7%. Gross total resection of the CCM was achieved in 100% of cases, with complete hemosiderin rim removal in 80% of applicable lesions. Early postoperative complications occurred in four patients, but at the maximum 48-month follow-up, 100% of the cohort achieved complete seizure control, and 44.4% successfully discontinued antiepileptic drugs. Long-term focal neurological deficits persisted in only two patients, while 77.8% were able to work following surgery. **Conclusions**: Microsurgical resection remains a well-established treatment modality for symptomatic CCMs. In this small, descriptive series of patients with lesions in functionally critical regions, high rates of gross total resection and favorable long-term seizure freedom were observed. These findings suggest that a multimodal approach combining anatomical neuronavigation with functional IONM may help minimize permanent morbidity, though larger cohorts are required to establish definitive efficacy.

## 1. Introduction

Cerebral cavernous malformations (CCMs) are vascular malformations made up of distended and loosely organized blood vessels, which generally lack neural tissue inside and smooth muscle and elastic fibers and are usually surrounded by hemosiderin and gliosis [1,2]. The majority of CCMs are supratentorial but can be found anywhere in the brain or spinal cord [1,3,4]. The prevalence of CCM is equal in men and women, with most being diagnosed between the 2nd and 4th decades [2]. CCMs can be sporadic and associated with developmental venous anomalies (DVAs) or can be familial autosomal dominant, driven by loss-of-function mutations in CCM1, CCM2, or CCM3 that disrupt endothelial junctional integrity, and those CCMs are usually multiple [5,6,7,8]. The clinical course is often characterized by progressive focal neurological deficits or refractory epilepsy, caused by the mass effect and the chemical irritation of the surrounding hemosiderin rim; alternatively, CCM can be an incidental finding on brain magnetic resonance imaging (MRI) performed for unrelated symptoms [2,6]. Conventional MRI with T1- and T2-weighted sequences is the modality of choice when it comes to diagnosing CCMs, as they are occult on digital subtraction angiography (DSA) and may not always be seen on computed tomography (CT) [9,10,11]. For lesions in eloquent brain regions, advanced modalities like functional MRI (fMRI) and diffusion tensor imaging (DTI)—particularly when tractography is integrated with intraoperative neuronavigation—are vital for preserving critical white matter tracts during potential microsurgical resection in cases of symptomatic CCMs [2,12,13]. As the likelihood of developing epilepsy after a first CCM-related seizure is high, patients require antiepileptic drugs (AEDs) [14]. Early microsurgical resection of the CCM and surrounding epileptogenic brain tissue represents a safe and effective therapy in cases of symptomatic CCMs [15,16,17]. When it comes to CCMs in eloquent brain areas, surgical management becomes challenging due to a high risk of neurological deficits after surgery [18]. Utilizing intraoperative imaging (ultrasound, neuronavigation, MRI), intraoperative neuromonitoring (IONM) (electrocorticography, motor and somatosensory evoked potentials) and intraoperative functional brain mapping at the cortical and subcortical levels, as well as performing surgical procedures under awake conditions, minimizes the surgical corridor’s impact on functionally intact brain parenchyma, thus making CCM resection surgery as safe and as effective as possible [19,20,21,22].

This article describes the surgical management of patients with symptomatic CCMs residing within eloquent brain regions, focusing on postoperative seizure control and functional outcomes. Utilizing a descriptive multicenter dataset, we aim to analyze clinical outcomes and report on the feasibility and observed results of microsurgical resection in a cohort of nine symptomatic patients, with specific emphasis on long-term seizure freedom and functional preservation. We also aim to highlight technical integration by demonstrating the utility and integration of intraoperative neurophysiological monitoring and advanced neuroimaging techniques in minimizing surgical morbidity. Additionally, this article provides a review of the current literature to provide a comprehensive discussion of contemporary surgical strategies and literature relevant to managing lesions in functionally critical areas.

## 2. Materials and Methods

We conducted a retrospective multicenter analysis of adult patients who underwent microsurgical resection for symptomatic CCMs located in eloquent brain regions between January 2021 and December 2025. The study was conducted at two tertiary referral centers in Slovenia: University Medical Center Maribor and University Medical Center Ljubljana.

### 2.1. Inclusion and Exclusion Criteria

Inclusion was limited to adult patients presenting with symptomatic CCMs—defined by the presence of focal neurological deficits or epilepsy (focal or generalized)—who underwent surgical treatment. Both hemorrhagic and non-hemorrhagic lesions were included, provided they were clinically symptomatic.

### 2.2. Definition of Eloquence

For the purposes of this study, eloquent cortical regions were defined as the primary motor, somatosensory, visual, and auditory cortices, as well as the primary language areas (Broca’s and Wernicke’s areas). Deep eloquent structures included the thalamus, hypothalamus, internal capsule, and brainstem.

### 2.3. Data Collection and Follow-Up

Clinical and demographic data were retrospectively retrieved from institutional electronic health records, operative reports, histopathological findings, and longitudinal radiological imaging. Postoperative follow-up was conducted through standardized neurological and neurosurgical outpatient evaluations.

### 2.4. Clinical Assessment

Clinical and functional statuses were evaluated preoperatively and during the postoperative follow-up period using standardized objective measures. Functional independence preoperatively and following surgery was assessed using the Karnofsky Performance Status scale. For patients presenting with epilepsy, postoperative seizure outcomes were evaluated and classified according to the International League Against Epilepsy (ILAE) outcome scale.

### 2.5. Graphical Abstract Design

The graphical abstract accompanying this manuscript was designed with the assistance of Gemini 3.5 Flash (Google LLC, Mountain View, CA, USA). The authors reviewed, edited, and finalized the output and take full responsibility for the final visual content.

### 2.6. Statistical Analysis

Due to the small sample size (*n* = 9) of this cohort, formal comparative statistical analysis and hypothesis testing were not performed. Instead, data were analyzed utilizing descriptive statistics. Continuous variables are expressed as means ± standard deviations (SDs) or as medians with ranges and interquartile ranges (IQRs), depending on data distribution. Categorical variables are presented as absolute frequencies and percentages (*n*, %).

## 3. Results

Between January 2021 and December 2025, nine patients (*n* = 9) underwent microsurgical resection for symptomatic CCMs within eloquent regions across two tertiary centers. The cohort included five females (55.6%) and four males (44.4%), with a mean age at diagnosis of 40.9 ± 14.5 years and a mean age at surgery of 44.1 ± 13.6 years. The median interval from diagnosis to surgical intervention was 37.8 months (range, 1–216 months); notably, one patient (Patient 6; 11.1%) underwent surgery 18 years after the initial diagnosis following a relocation from abroad.

Seizures were the primary clinical presentation in seven out of nine cases (77.8%), with six patients (66.7%) experiencing focal seizures and one patient (11.1%) experiencing generalized seizures. The latter patient also presented with Gerstmann’s syndrome. In two cases (22.2%), patients’ symptoms were non-epileptic: one patient (11.1%, Patient 4) presented with diplopia and ataxia, while one patient (11.1%, Patient 5) exhibited hemiparesis and hemisensory deficits, reflecting the involvement of their respective eloquent regions. None of the patients (0%) had a history of cranial radiotherapy, familial CCM syndromes, or prior cavernoma-related interventions. Detailed patient characteristics are summarized in Table 1.

Of the seven patients presenting with seizures (*n* = 7), four (57.1%) demonstrated radiological evidence of acute hemorrhage within the CCM, while three (42.9%) presented with seizures in the absence of hemorrhage. Preoperative focal neurological deficits were present in four patients (44.4%): Patient 1 presented with Gerstmann’s syndrome; Patient 4 exhibited right-eye adduction palsy, bidirectional horizontal nystagmus, left-arm dysmetria/ataxia, broad-based gait, and diplopia; Patient 5 presented with flaccid right-sided hemiparesis, anisocoria, and a right-sided hemisensory deficit; and Patient 9 presented with monoparesis of the upper extremity. Conversely, five patients (55.6%) had no preoperative focal neurological deficits. In the observed cohort (*n* = 9), two patients (22.2%) did not suffer from seizures, one (11.1%) had a single seizure, and the majority (six patients, 66.7%) suffered from refractory seizures. Preoperatively, six patients (66.7%) were managed with a single AED, and one patient (11.1%) required dual-agent therapy. Patient 4 (11.1%) received prophylactic AED therapy despite the absence of seizures. Surgical intervention was primarily indicated due to refractory epilepsy in three patients (33.3%) with non-hemorrhagic CCMs. In the remaining six cases (66.7%), surgery was indicated due to acute hemorrhage and the associated risk of recurrent bleeding; this included Patient 1 (11.1%), whose seizures were controlled by medication. Notably, Patient 7 (11.1%) underwent resection during pregnancy following a symptomatic bleed for fear of a recurrent hemorrhaging event. Detailed clinical characteristics and indications are demonstrated in Table 2.

All patients in the observed cohort (*n* = 9, 100%) underwent MRI for the diagnosis of CCMs and were evaluated by a neurologist for AED management. Following the identification of refractory seizures or hemorrhage on neuroimaging, a neurosurgical consultation was obtained to discuss operative resection. Based on radiological and clinical findings, five patients (55.6%) presented with a single hemorrhage within the CCM, while one patient (11.1%, Patient 5) experienced three distinct bleeding episodes. In one case (11.1%, Patient 9), multiple cavernomas were present, though resection was strictly indicated for the single lesion demonstrating acute hemorrhage. Regarding advanced preoperative diagnostics, seven patients (77.8%) underwent fMRI, three (33.3%) additionally underwent MRI tractography, five (55.6%) underwent electroencephalography (EEG), and three (33.3%) received formal neuropsychological testing. Prior to surgery, four patients (44.4%) were actively employed, four (44.4%) were unable to work due to neurological morbidity, and one patient (11.1%) was retired. The median preoperative Karnofsky Performance Status (KPS) score was 90 (range, 30–100; interquartile range [IQR], 70–100). Detailed preoperative workup and baseline characteristics are provided in Table 3.

Lesions were predominantly left-sided (seven patients, 77.8%) and located across various eloquent zones: four were deep-seated or brainstem/posterior fossa lesions—specifically, two (22.2%) were deep-seated, one (11.1%) was insular, and one (11.1%) was in the left cerebellar peduncle—while the remainder (five patients, 55.6%) were lobar. The mean maximal CCM diameter was 19 mm (range, 9–27 mm), with a mean volume of 4.76 mL (range, 0.38–10.3 mL). A hemosiderin rim was radiologically evident in five patients (55.6%). For these lesions, the mean total diameter including the hemosiderin rim was 25.6 mm (range, 16–31 mm), with a mean total volume of 5.07 mL (range, 0.99–11.56 mL). A detailed presentation of cavernoma characteristics is provided in Table 4.

Intraoperatively, a neuronavigation system was utilized in all nine cases (*n* = 9, 100%), and intraoperative ultrasound (US) was additionally employed in five cases (55.6%). To preserve functional integrity, two patients (22.2%, Patients 1 and 3) underwent awake craniotomy for lesions involving eloquent language areas (parietotemporal and insular, respectively), while the remaining seven patients (77.8%) underwent surgery under general anesthesia. IONM—including motor evoked potentials (MEPs) and somatosensory evoked potentials (SSEPs)—was employed in seven cases (77.8%), with cortical mapping specifically applied in all seven of those instances (100% of monitored cases). Gross total resection of the CCM core was achieved in all nine patients (100%), as confirmed by both intraoperative assessment and postoperative MRI. Among the patients presenting with a hemosiderin rim (*n* = 5), complete rim resection was achieved in four cases (80.0%); resection of the rim was intentionally limited in one case (20.0%, Patient 3) to avoid exacerbating transient sensory speech deficits. A detailed analysis of intraoperative modalities and the extent of resection is presented in Table 5.

Early postoperative complications were noted in four patients (44.4%), while five patients (55.6%) experienced an uneventful early postoperative course. Among those with complications, Patient 2 (11.1%) developed left-sided hemiparesis following an intraoperative loss of MEPs, Patient 7 (11.1%) developed mild right foot paresis (with no obstetric complications reported), and Patient 8 (11.1%) experienced transient right oculomotor nerve palsy. Additionally, Patient 3 (11.1%) developed a surgical site infection 3 months postoperatively that required a return to the operating room for debridement and a course of intravenous antibiotics; however, they completely recovered without neurological sequelae.

The first postoperative follow-up occurred 3 to 6 months after surgery, with a maximum follow-up duration of 48 months. The median postoperative follow-up period was 8 months (range, 3–48 months; IQR, 6–12 months). At the latest follow-up, complete seizure control was achieved in all seven patients who had presented with preoperative epilepsy (100% seizure-freedom rate among affected patients). Five patients (55.6% of the total cohort) were still receiving AED therapy at the last follow-up: four (44.4%) on monotherapy and one (11.1%, Patient 8) on dual-agent therapy. Conversely, four patients (44.4%) were entirely AED-free, including three (33.3%) who had presented with preoperative epilepsy.

Long-term motor deficits persisted in two patients (22.2%, Patients 2 and 7), while no patients (0%) exhibited long-term speech deficits. Socioeconomic outcomes at the final follow-up revealed that seven patients (77.8%) were able to work in their profession, one patient (11.1%) was retired, and one patient (11.1%, Patient 2) was unable to work due to persistent postoperative hemiparesis. Overall, the cohort demonstrated stable to improved functional outcomes. While the median preoperative KPS score was 90 (range, 30–100; IQR, 70–100), the median postoperative KPS score remained 90, but the lower bound of the cohort improved, yielding a postoperative range of 60–100 (IQR, 80–90). All nine patients (100%) remain under regular clinical follow-up by their attending neurologist and/or neurosurgeon. Detailed long-term outcomes are summarized in Table 6.

## 4. Discussion

While representing a limited series of nine patients, our findings suggest that microsurgical resection supported by appropriate intraoperative adjuncts remains a valuable and effective approach for highly selected, eloquent CCMs, providing favorable clinical and seizure outcomes. In our small cohort, where seizures were the primary clinical presentation (77.8%), we achieved complete seizure control (ILAE Class 1) and gross total resection in all cases. Our total hemorrhage rate was 66.7%, reflecting a highly symptomatic patient selection necessitated by the critical, eloquent location of these lesions. Given the small sample size, these descriptive metrics cannot be widely generalized, but they offer valuable insights into the management of high-risk eloquent lesions.

This highly symptomatic profile is noteworthy when compared to the large international survey by Zanello et al. [18], which involved 272 patients and reported lower baseline rates of symptomatic hemorrhage (48.7%) and seizures (53.9%). To manage these high-risk lesions, our integration of intraoperative technology was notably robust. Neuronavigation was utilized in 100% of cases and intraoperative ultrasound (US) in 55.6%, compared to 91.5% and 32.1% in the Zanello survey, respectively. Furthermore, our use of IONM with MEPs/SSEPs (77.8% vs. 27%) and cortical mapping (77.8% vs. 58.1%) exceeded international averages. These adjuncts were instrumental in achieving GTR in our nine patients (compared to 97.8% in Zanello et al.), proving particularly helpful during awake craniotomies in dominant-hemisphere speech centers where speech function was preserved.

Total resection of the hemosiderin rim—a key driver of post-cavernoma epilepsy—was achieved in 80% of applicable cases (vs. 71% in the Zanello survey). Rim resection was limited in only one instance due to transient intraoperative speech disturbances, underscoring the delicate balance between aggressive resection and functional preservation. Clinically, 100% of the epileptic cohort achieved seizure freedom, with 42.8% successfully discontinuing antiepileptic drug (AED) therapy. While these early results are encouraging, the small cohort size and variable follow-up duration (median: 8 months; range: 3–48 months) mean that outcomes—especially for individuals followed for less than 6 months—must be treated as preliminary. Extended longitudinal tracking of a larger patient population is required to fully validate the durability of these outcomes.

Functional and socioeconomic recovery in our cohort was favorable. Preoperatively, 44.4% of patients were unable to work (vs. 22.7% in Zanello et al.) [18], whereas postoperatively, 77.8% returned to work. Only one patient remained unable to work due to a new, persistent hemiparesis, yielding a postoperative work inability rate (11.1%) comparable to that in the Zanello series (11.8%). Similarly, Karnofsky Performance Status (KPS) scores demonstrated overall stability, with the minimum cohort score rising from 30 to 60, reflecting clinical improvement in the most severely symptomatic patients.

The natural history of CCMs underscores the necessity of considering intervention. Previous hemorrhage is an established risk factor for recurrent bleeding, with deep or brainstem locations further compounding this risk [23,24]. Additionally, CCM-related hemosiderin deposition induces surrounding gliosis, leading to drug-resistant epilepsy (DRE) in up to 40% of cases [14,25]. While the literature debates the necessity of extended lesionectomy [26,27], our strategy prioritizing the clearance of both the lesion and accessible surrounding hemosiderin aligns with data showing that this approach can be sufficient for seizure control [28,29]. The therapeutic value of extended lesionectomy is strongly supported by large-scale data. In a major meta-analysis of 594 patients by Ruan et al. [15], complete excision of the perilesional hemosiderin rim yielded a significantly higher rate of long-term seizure freedom compared to simple lesionectomy alone (74% vs. 68%; OR = 0.62, *p* = 0.01). This is echoed by Hammen et al. [30], who noted that incomplete rim resections directly correlated with persistent postoperative seizures, though they highlighted that a shorter pre-surgical epilepsy history optimizes outcomes.

However, because these studies evaluate mixed cohorts, their aggressive parameters must be adapted when operating strictly within eloquent brain regions. By using real-time IONM and awake mapping to halt resection at the exact boundary where epileptogenic iron staining meets functioning neural tracts, we successfully balanced maximal seizure control with strict functional preservation. This tailored approach explains why rim resection was intentionally limited in only one instance in our series (Patient 3) due to transient intraoperative speech disturbances, successfully preserving long-term neurological function while still achieving ILAE Class 1 seizure freedom.

Navigating eloquent regions demands a clear technical strategy. While imaging-based modalities like neuronavigation provide essential structural frameworks, they remain vulnerable to intraoperative brain shift [31,32]. Therefore, functional mapping via electrical brain stimulation and real-time IONM are indispensable for guiding corticectomies, protecting critical white matter tracts, and preserving associated developmental venous anomalies (DVAs) [15,31]. While non-invasive alternatives like stereotactic radiosurgery (SRS) and MRI-guided laser interstitial thermal therapy (LITT) exist for deep, surgically inaccessible lesions [2,33], open microsurgery remains the primary choice in our series to achieve immediate mass-effect relief and complete lesion elimination.

In conclusion, indicating surgery for eloquent CCMs requires a high threshold due to potential deficits, and our study is inherently underpowered to definitively establish generalized safety and efficacy guidelines. However, driven by drug-resistant focal epilepsy and high hemorrhage risk, a multimodal approach combining structural and functional intraoperative adjuncts successfully mitigated these hazards in our series. This synergy allowed for aggressive lesion and rim removal, offering the patients in this limited cohort a seizure-free life and protection against recurrent hemorrhage.

### Study Limitations

We acknowledge several limitations inherent to this study. Primarily, its retrospective design and small sample size (*n* = 9) restrict broader statistical generalizability and preclude the calculation of statistical significance regarding surgical risk factors, outcome predictors, or the comparative efficacy of specific intraoperative adjuncts. Consequently, our findings represent descriptive institutional trends rather than definitive clinical guidelines. Additionally, the anatomical heterogeneity of the lesion locations within our cohort—spanning distinct eloquent zones such as language centers, motor pathways, and deep structures—creates diverse surgical demands and limits direct, uniform comparisons with larger, more homogeneous published series.

Moreover, because our cohort is exclusively surgical and lacks a conservatively managed comparison group, it is subject to institutional referral bias. As two specialized tertiary referral centers, our registry naturally captures highly symptomatic cases, accounting for the high baseline prevalence of aggressive hemorrhages and drug-resistant epilepsy. Finally, the wide variation in postoperative follow-up (median: 8 months; range: 3–48 months) introduces chronological constraints. While patients with extended tracking demonstrated sustained stability, outcomes for those with shorter follow-up windows (e.g., <6 months) remain preliminary, necessitating continued long-term surveillance to confirm the durability of seizure freedom.

## 5. Conclusions

Microsurgical resection remains a widely accepted treatment strategy for symptomatic CCMs. The successful management of lesions in eloquent regions often relies on a tailored approach using IONM, brain mapping, and neuronavigation to optimize the extent of resection while minimizing permanent neurological deficits. Within our retrospective series, the integration of these intraoperative technologies was associated with complete resection and favorable symptom management. While the descriptive nature and limited sample size of our study preclude definitive conclusions regarding safety and efficacy, our observations align with the broader literature—such as the findings by Zanello et al. [18]—supporting the utility of a multimodal surgical approach in eloquent areas.

## Figures and Tables

**Table 1:** Baseline clinical and demographic characteristics of the study cohort (*n* = 9).

Patient ID	Gender	Age at Diagnosis (Years)	Age at Surgery (Years)	Time from Diagnosis to Surgery (Months)	CCM Location	Primary Clinical Presentation
**1**	Male	33	34	11	Left parietotemporal region	Seizures, Gerstmann syndrome
**2**	Male	56	57	12	Basal ganglia	Refractory focal seizures
**3**	Female	31	31	1	Insular region	Focal seizures
**4**	Female	37	37	1	Left cerebellar peduncle	Diplopia, ataxia
**5**	Female	36	41	48	Thalamus	Hemiparesis, hemisensory deficits
**6**	Male	27	45	216	Parietotemporal region	Refractory focal seizures
**7**	Female	29	29	1	Parietal region	Focal seizures (during pregnancy)
**8**	Female	49	53	48	Medial temporal lobe	Refractory focal seizures
**9**	Male	70	70	2	Parietal lobe	Refractory focal seizures, upper extremity monoparesis

**Table 2:** Preoperative AED management and neurological status (*n* = 9).

Patient ID	Mode of Clinical Presentation	Preoperative Focal Deficit	Seizure Frequency Prior to Surgery	AED Regimen at Time of Surgery	Seizure Control Status
**1**	Hemorrhage & Seizure	Yes	Single seizure	Levetiracetam monotherapy	Controlled
**2**	Seizure	No	Multiple (>1)	Brivaracetam + Lamotrigine	Refractory
**3**	Hemorrhage & Seizure	No	Multiple (>1)	None	Refractory (Untreated)
**4**	Hemorrhage	Yes	0	Levetiracetam (Prophylactic)	No history of seizures
**5**	Hemorrhage	Yes	0	None	No history of seizures
**6**	Seizure	No	Multiple (>1)	Carbamazepine monotherapy	Refractory
**7**	Hemorrhage & Seizure	No	Multiple (>1)	Levetiracetam monotherapy	Refractory
**8**	Seizure	No	Multiple (>1)	Lamotrigine monotherapy	Refractory
**9**	Hemorrhage & Seizure	Yes	Multiple (>1)	Levetiracetam monotherapy	Refractory

**Table 3:** Preoperative bleeding history and functional performance status (*n* = 9).

Patient ID	Preoperative Hemorrhagic Events (*n*)	Preoperative KPS Score	Preoperative Employment Status
**1**	1	90	Employed
**2**	0	70	Unemployed (due to illness)
**3**	1	100	Employed
**4**	1	60	Unemployed (due to illness)
**5**	3	30	Unemployed (due to illness)
**6**	0	100	Employed
**7**	1	100	Employed (pregnant at time of surgery)
**8**	0	70	Unemployed (due to illness)
**9**	1	90	Retired

**Table 4:** Radiological and morphological characteristics of CCMs (*n* = 9).

Patient ID	Lesion Location & Lateralization	Maximal Core Diameter (T1, mm)	Core Volume (T1, mL)	Hemosiderin Rim Present	Maximal Rim Diameter (T2, mm) *	Total Rim Volume (T2, mL) *
**1**	Left parietotemporal lobe	19	3.59	Yes	22	1.98
**2**	Right deep-seated (basal ganglia)	13	1.15	Yes	16	0.99
**3**	Left insular cortex	17	2.57	Yes	30	11.56
**4**	Left cerebellar peduncle	23	6.37	No	—	—
**5**	Left deep-seated (thalamus)	9	0.38	No	—	—
**6**	Right parietotemporal lobe	27	10.30	Yes	29	2.46
**7**	Left parietal lobe	24	7.24	Yes	31	8.36
**8**	Left temporal lobe (parahippocampal)	12	0.90	No	—	—
**9**	Left parietal lobe	27	10.30	No	—	—

Note: * Values represent measurements including the surrounding hemosiderin halo where applicable. Em-dashes (—) indicate absence of a radiologically distinct hemosiderin rim.

**Table 5:** Intraoperative modalities and extent of surgical resection (*n* = 9).

Patient ID	Intraoperative Modalities Employed	Extent of CCM Core Resection	Extent of Hemosiderin Rim Resection
**1**	Neuronavigation, US, Awake Craniotomy, IONM (MEP/SSEP)	GTR	Complete
**2**	Neuronavigation, US, IONM (MEP/SSEP)	GTR	Complete
**3**	Neuronavigation, US, Awake Craniotomy, IONM (MEP/SSEP)	GTR	Partial (Intentionally limited)
**4**	IONM (MEP/SSEP)	GTR	— *(No preoperative rim)*
**5**	Neuronavigation, IONM (MEP/SSEP)	GTR	— *(No preoperative rim)*
**6**	Neuronavigation, US, IONM (MEP/SSEP)	GTR	Complete
**7**	Neuronavigation, US, IONM (MEP/SSEP)	GTR	Complete
**8**	Neuronavigation	GTR	— *(No preoperative rim)*
**9**	Neuronavigation	GTR	— *(No preoperative rim)*

Abbreviations: GTR, gross total resection; IONM, intraoperative neurophysiological monitoring; US, ultrasound; MEP, motor evoked potentials; SSEP, somatosensory evoked potentials.

**Table 6:** Postoperative seizure control, AED therapy, and functional status at final follow-up (*n* = 9).

Patient ID	Seizure Outcomes (ILAE Class) *	AED Status	Specific Postoperative AED Regimen	Postoperative KPS Score
**1**	Class I	Off AEDs	—	100
**2**	Class I	On AEDs	Brivaracetam monotherapy	60
**3**	Class I	Off AEDs	—	100
**4**	—	Off AEDs	—	80
**5**	—	On AEDs	Levetiracetam monotherapy (Prophylactic)	80
**6**	Class I	On AEDs	Carbamazepine monotherapy	90
**7**	Class I	Off AEDs	—	90
**8**	Class I	On AEDs	Brivaracetam + Lamotrigine (Dual therapy)	90
**9**	Class I	On AEDs	Levetiracetam monotherapy	90

Note: * ILAE Class I denotes complete seizure freedom since surgery. Em-dashes (—) for Patients 4 and 5 indicate that these patients did not present with preoperative seizures/epilepsy. Abbreviations: AED, antiepileptic drug; ILAE, International League Against Epilepsy; KPS, Karnofsky Performance Status.

## Data Availability

Data is contained within the article.

## Abbreviations

The following abbreviations are used in this manuscript:

CCMs	Cerebral cavernous malformations
DVAs	Developmental venous anomalies
MRI	Magnetic resonance imaging
DSA	Digital subtraction angiography
CT	Computed tomography
fMRI	Functional magnetic resonance imaging
DTI	Diffusion tensor imaging
AEDs	Antiepileptic drugs
IONM	Intraoperative neuromonitoring
EEG	Electroencephalogram
US	Ultrasound
MEPs	Motor evoked potentials
SSEPs	Somatosensory evoked potentials
DRE	Drug-resistant epilepsy
SRS	Stereotactic radiosurgery
GKRS	Gamma knife radiosurgery
LITT	Laser interstitial thermal therapy
